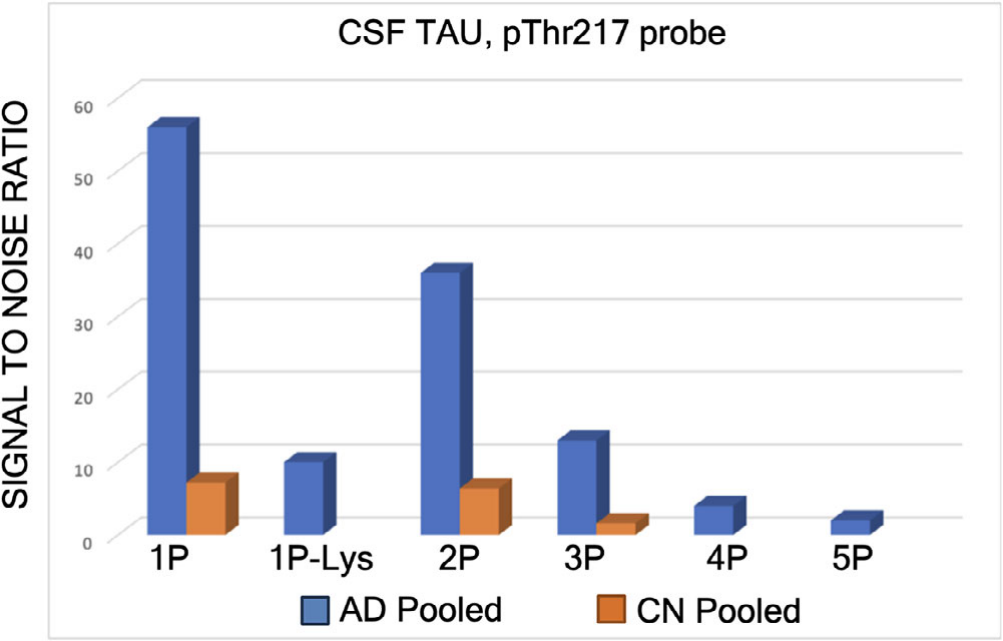# Targeted Multiplexed MALDI MS Platform Enables Detection of Novel Pathogenic Forms of Tau, Amyloid Beta and Alpha Synuclein

**DOI:** 10.1002/alz.087292

**Published:** 2025-01-09

**Authors:** Vladislav Bergo

**Affiliations:** ^1^ Adeptrix Corp, Beverly, MA USA

## Abstract

**Background:**

The rapidly growing pipeline of target‐specific Alzheimer’s Disease (AD) therapeutic candidates requires accompanying tests that can identify patients likely to have a beneficial response. The growing importance of multiple pathologies in determining AD progression and treatment response underscores this need. Our work focuses on establishing analytical capability to expand detectable forms of major protein drug targets for AD: Tau, amyloid beta (Ab) and a‐Synuclein (aS) proteoforms as potential personalized molecular signatures.

**Method:**

Novel analytical workflow (BAMS™) based on single bead immunoaffinity capture, enrichment and mass spectrometry (MS) quantification was applied to measure distinct species of CSF Tau, Aβ and αS. Samples from cognitively normal (CN) and AD subjects were acquired from a biobank and measured either non‐digested or after digestion with LysC protease. MS data was collected on Bruker Autoflex Max MALDI TOF instrument and processed using in‐house developed software.

**Result:**

BAMS™ has detected >40 CSF Aβ peptides, comprising N‐terminal Aβ_X‐40/42_ (X= 1, 3, 5, 7, 10, 11) and C‐terminal Aβ_1‐X_ (X between 6 – 43) truncation series. The latter included previously unreported peptides Aβ_1‐6_‐Aβ_1‐11_, Aβ_1‐21_‐Aβ_1‐26_, Aβ_1‐31_ and Aβ_1‐32_. Many peptides including amyloid plaque pathology markers Aβ_1‐40_ and Aβ_1‐42_ were measured with CVs under 10%. A survey of CN and AD subjects reveals major differences within Aβ_1‐X_ patterns likely reflecting different amyloid metabolism. Total and phospho‐Tau (tTAU and pTAU) probes revealed extensive endogenous proteolysis in the Proline Rich Domain with multiple detected fragments terminating at distinct proline sites. The pTAU probe detected hyperphosphorylated sequences containing up to 3 and 5 modified sites in CN and AD, respectively, which unambiguously differentiated normal from diseased samples. Three αS probes have been developed that measure: (1) total protein abundance, (2) C‐terminal fragmentation and (3) relative abundance of monomer and dimer species of isoforms SNCA140, SNCA126 and SNCA112.

**Conclusion:**

Healthy and diseased human CSF may contain a wider range of Tau, Aβ and αS proteoforms than previously recognized. BAMS™ can simultaneously measure multiple protein targets and multiple proteoforms via independent probing of distinct protein regions. The broad sequence coverage enables screening of multiple biomarker candidates for accurate molecular profiling of tau tangles, amyloid plaques and Lewy bodies.